# Effects of Psychrophilic Storage on Manures as Substrate for Anaerobic Digestion

**DOI:** 10.1155/2014/712197

**Published:** 2014-08-05

**Authors:** Wenche Bergland, Carlos Dinamarca, Rune Bakke

**Affiliations:** Department of Process, Energy and Environmental Technology, Telemark University College, 3918 Porsgrunn, Norway

## Abstract

The idea that storage can enhance manure quality as substrate for anaerobic digestion (AD) to recover more methane is evaluated by studying storage time and temperature effects on manure composition. Volatile fatty acids (VFA) and total dissolved organics (CODs) were measured in full scale pig manure storage for a year and in multiple flasks at fixed temperatures, mainly relevant for colder climates. The CODs generation, influenced by the source of the pig manure, was highest initially (0.3 g COD L^−1^d^−1^) gradually dropping for 3 months towards a level of COD loss by methane production at 15°C. Methane emission was low (<0.01 g COD L^−1^d^−1^) after a brief initial peak. Significant CODs generation was obtained during the warmer season (T > 10°C) in the full scale storage and almost no generation at lower temperatures (4–6°C). CODs consisted mainly of VFA, especially acetate. All VFAs were present at almost constant ratios. The naturally separated manure middle layer without sediment and coarser particles is suitable for sludge bed AD and improved further during an optimal storage time of 1–3 month(s). This implies that high rate AD can be integrated with regular manure slurry handling systems to obtain efficient biogas generation.

## 1. Introduction

Anaerobic digestion of manure can reduce greenhouse gas emissions (GHGE) and odors, produce renewable energy in the form of biogas, and enhance manure fertilizer quality [1]. The largest potential source of methane by AD of wet organic waste is manure, for example, ~40% in Norway; however, only a small fraction of this is realized [2]. The main reason for this is the low energy density of manure, implying low production rates in continuous flow stirred tank reactors (CSTR) currently used for manure AD. Such solutions are not economically sustainable in Norway because the costs of construction and operation of such plants are larger than the value of the methane produced [2]. Some large scale farms have their own CSTR AD solutions that are economically sustainable, for example, in Denmark [3], but agriculture in Norway is dominated by smaller farms where such systems are not profitable [2]. It is assumed that small farms constitute a large fraction of global agriculture also, so that the “Norwegian case” investigated here has international relevance. Manure transport to central AD treatment is used to some extent, especially in Germany, but the sustainability of such solutions is questioned mainly due to transport cost of manure with low biogas potential and greenhouse gas emissions [4]. New process solutions for AD treatment of manure must therefore be developed to realize the enormous total energy potential of this source.

High rate AD (HRAD) reactors may solve this problem by treating more waste in smaller and presumably much cheaper digesters. AD manure treatment that is well integrated with existing farm infrastructure for liquid (slurry) based manure handling is therefore suggested and evaluated here as a strategy for cost-effective biogas generation. Liquid based manure handling systems are common for cattle and pig farms [5] where all excreta are collected in liquid form with some dilution from wash water. Manure from farms using liquid/slurry based handling systems has, for example, 61% of the total theoretical Norwegian manure energy potential of 2480 GWh/a [6]. Manure storage tanks with 8 months minimum hydraulic retention time (HRT) capacity are included in existing farm infrastructure in cold climate countries (e.g., Norway, to comply with government regulations to avoid pollution and use as fertilizer during the short growth season only). Such storage facilities may serve as the first step in an AD treatment line and/or be used for AD effluent storage in combination with HRAD. Existing pig slurry storage has uncontrolled methane release [7] so treating such slurries by harvesting and using methane has the additional environmental benefit of reducing GHGE from slurry storages.

**Figure 1:**
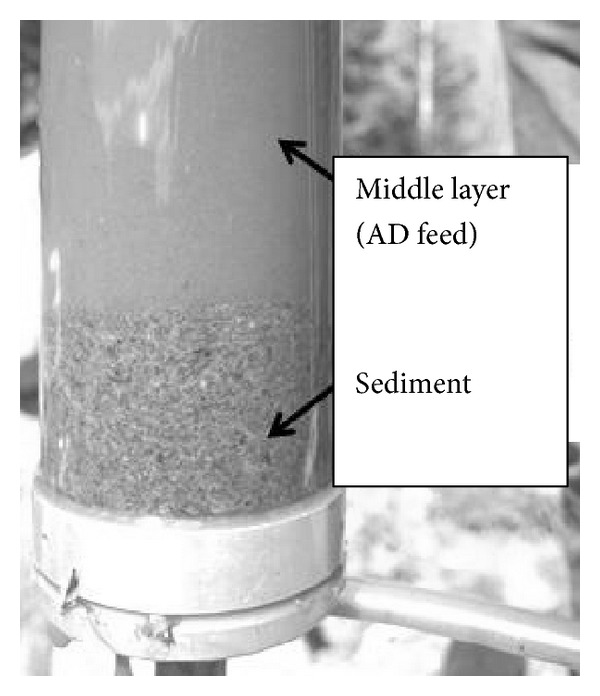
Pig manure sample collected near the bottom of a pig manure storage tank, showing the distinct interface between the sediment and the middle layer.

It has been observed that manure particles disintegrate and hydrolyze during storage [8], thereby improving its quality as AD feed. Examining in greater detail the manure changes during storage is carried out here to evaluate how well manure storage can serve as a first step in an AD treatment line. It has also been observed that manure separates into a floating layer (straw, wood chips, etc.), a bottom sediment layer, and a middle layer with much less suspended solids than the floating and bottom layers. Pig manure separates spontaneously into such distinct layers as seen in Figure 1, implying that potentially suitable high rate AD feed can be taken out from the middle layer at no extra cost. The middle layer may not be best for AD in general, but it is best for sludge bed based HRAD since such reactors require a feed with relatively low particle content and/or low viscosity to avoid losing the culture by flushing out the sludge bed [9].

Several degradation processes, such as those included in the anaerobic digestion model ADM1 [10], can occur during manure storage that can influence the quality of the manure as feed for AD and emissions during storage. The hypothesis tested here is that there is an optimal storage time that depends on the storage temperature. This is based on the assumption that biogas yield will increase if the manure is stored before AD since this will allow particle disintegration and large molecules to hydrolyze into dissolved monosaccharides, amino acids, long chain fatty acids, and VFA that can be converted to methane when used as feed for AD. It is also expected that such easily degradable organic molecules will be degraded all the way to methane in the storage if allowed too long storage.

The aim of the study is to identify an optimal time range for manure storage prior to AD as a function of temperature. The main focus is on Nordic (psychrophilic) conditions including summer temperatures. The evaluation is based on measurements of dissolved organics and methane yield.

## 2. Materials and Methods

The properties of manure from a pig production farm in southern Norway, Porsgrunn (59.2°N, 9.7°E) were examined during storage. The farm has three production stages/areas: “Sows,” “Growers,” and “Farrow and Wieners.” All animals are fed protein concentrate (14.6% crude protein) added to some grass/straw. The pig production unit uses wood shavings and straw as bedding material. Manure was examined both at controlled temperature conditions and in a storage basin at the farm during one year.

### 2.1. Sample Collection and Testing Scheme

Manure from the production stage Farrow and Wieners was collected from the manure channel in the barn and stored under controlled conditions at 11°C, 15°C, and 20–23°C for 3 months to monitor the content of easily degradable organics in the liquid manure. 100 mL infusion glass bottles with rubber stopper and metal ring were used. One bottle stored at each temperature was terminated regularly to analyze the liquid content. One bottle was used as parallel for each temperature case, with a total of 17 bottles. Syringe needles were placed through the stoppers of these 17 bottles to release produced biogas.

Manure from all 3 production stages was collected, sieved through a 2 mm sieve, and stored in 54 (100 mL) infusion glass bottles with rubber stopper and metal ring under controlled conditions at 15°C for maximum 4 months to study the effect of the pig production stage on manure development. Three parallel bottles from each pig production stage were terminated regularly to analyze the liquid content. Syringe needles were placed through the stoppers of these 54 bottles to release produced biogas. Sieved manure samples from the 3 production stages were also studied in 1000 mL infusion glass bottles for biogas production monitoring since not enough biogas for volume and composition measurements was produced in the smaller bottles. Syringe needles were placed through the stoppers of these 9 bottles and syringes were connected to the needles to collect biogas samples, to measure volume and composition of the produced biogas.

To evaluate the amount of methane potentially released from the long time storage, the methane potential (*B*_0_) of the sieved manure was measured via volume displacement using 3 parallels of 100 mL medical syringes with 2 mL gradations while stored at 35°C. 30 mL manure (with no inoculum) was placed in each syringe and the biogas production was read regularly, directly as the syringe piston displacement. When enough gas was produced the syringes were emptied and the gas composition measured.

### 2.2. Manure Handling and Examination at the Farm

Manure handling at the farm involves first manually pushing manure into channels in the floor twice a day, before it is pumped to a 300 m^3^ farm building basement storage basin; ~1/3 of the manure comes from each of Farrows and Wieners, Sows, and Growers. The basin manure also contains ~5% of wash water from regular barn washing routines. The content in the basin storage is stirred regularly in order to pump half of the basin volume content each time further to a 1600 m^3^ outdoor storage. This gives an average 50 d HRT in the basement storage basin.

Samples were siphoned from the liquid middle layer (Figure 1) during the whole year of 2012. 10–80 liters were collected each time. Temperature was measured in the collected sample immediately after removal from the storage.

### 2.3. Analysis

Total COD (COD_T_), soluble COD (COD_S_), total solids (TS), volatile solids (VS), pH, alkalinity, NH_4_^+^-N, VFAs (acetate, propionate, butyrate, isobutyrate, valerate, and isovalerate), and gas composition were analyzed.

COD, TS, VS, and alkalinity were measured according to US standard 5220D, 2540D, and 2320B, respectively [11]. For CODs determination the samples were first centrifuged at 10000 rpm for 30 minutes and then filtered (0.45 *μ*m). NH_4_^+^-N concentration was analyzed on filtered samples (0.2 *μ*m) by ion chromatography using a DX-500 ion chromatographic analyzer equipped with a conductivity detector, a SCS1 cation-exchange column (4 × 250 mm) in combination with a Dionex IonPac PCG1 (4 × 50 mm) guard column; 4 mM methane-sulfonic acid was used as the mobile phase. The oven temperature was kept constant at 35°C. VFA were measured by gas chromatography (Hewlett Packard 6890) with a flame ionization detector and a capillary column (FFAP 30 m, inner diameter 0.250 mm, and film 0.5 *μ*m). The oven was programmed to go from 100°C, hold for one minute, to 200°C at a rate of 15°C min^−1^ and then to 230°C at a rate of 100°C min^−1^. The carrier gas used was helium at 23 mL min^−1^. The injector and detector temperatures were set to 200°C and 250°C, respectively. Gas composition (CO_2_ and CH_4_) was quantified by gas chromatography (Hewlett Packard 5890A) equipped with a thermal conductivity detector and two columns connected in parallel: Column 1, CP-Molsieve 5A (10 m × 0.32 mm), and Column 2, CP-PoraBOND Q (50 m × 0.53 mm). The gas carrier was argon at 3.5 bar pressure. The oven temperature was kept constant at 40°C.

## 3. Results and Discussion

### 3.1. Farrow and Wieners Manure Storage Test at 3 Temperatures

**Figure 2:**
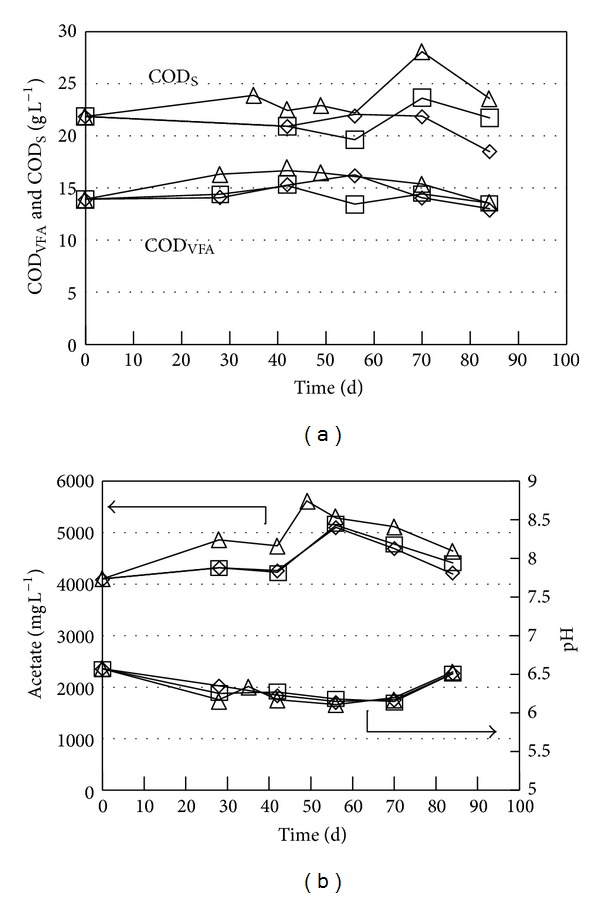
Acetate, COD_VFA_, pH, and COD_S_ during storage for Farrow and Wieners manure: 11°C (◊), 15°C (□), and 20–23°C (∆).

Only a slight increase in acetate (maximum 30% increase) and total VFA (maximum 20% increase) were observed during storage of Farrow and Wieners manure at 11°C, 15°C, and 20–23°C (Figure 2). No difference in VFA production was observed between 11°C and 15°C, but higher acetate and total VFA concentrations were obtained at 20–23°C. The pH went quickly from 6.5 to 6.2-6.3 at all three temperatures. The pH changes can be explained by both produced VFA and CO_2_ [12]. There appears to be a small temperature effect on COD_S_ with a slight increase at the highest temperature, but all changes are in the range of the standard deviation and therefore not considered as significant. Storage time and temperature of the manure from Farrow and Wieners have therefore little effect on the quality of, for example, AD feed. The low pH implies that there is little risk of methanogenesis and loss of NH_3_ during storage of this manure fraction. It may therefore be stored and used for biogas production on demand.

### 3.2. Comparison between the Pig Production Stages at 15°C

#### 3.2.1. Liquid Properties

**Figure 3:**
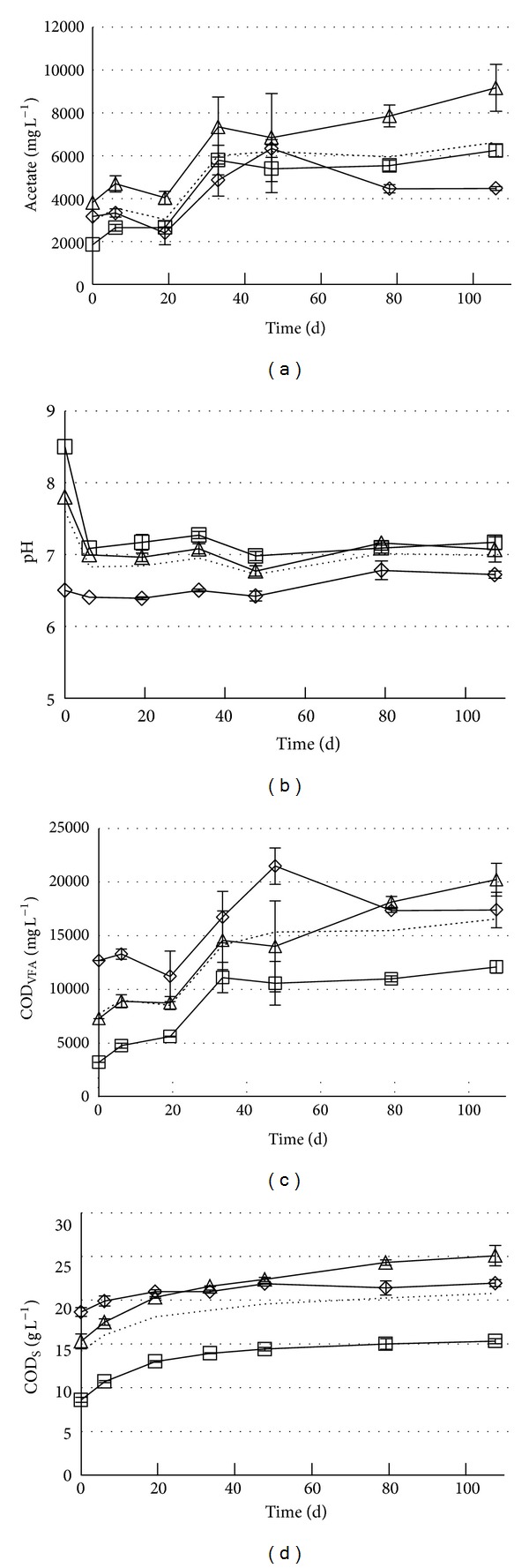
Acetate, COD_VFA_, pH, and COD_S_ during storage at 15°C: Sows (□), Growers (∆), and Farrow and Wieners (◊), average–dotted line.

Pronounced differences between the manures from the different pig production stages in the way their compositions changed with time were observed when they were all stored at 15°C (Figure 3). The least changes were observed during storage of the manure from the Farrow and Wieners, even though it had the highest initial concentrations of both COD_S_ and COD_VFA_ and also the lowest initial pH. Manure from Growers had the highest levels of acetate, COD_S_ (after 30–40 days of storage), and COD_VFA_ (after 78 days of storage). Manure from Sows had the lowest concentration of soluble organics both as COD_S_ and COD_VFA_ and also the highest pH. Manure from Sows is therefore expected to give less methane yield than manure from the other stages when used as substrate for AD.

**Figure 4:**
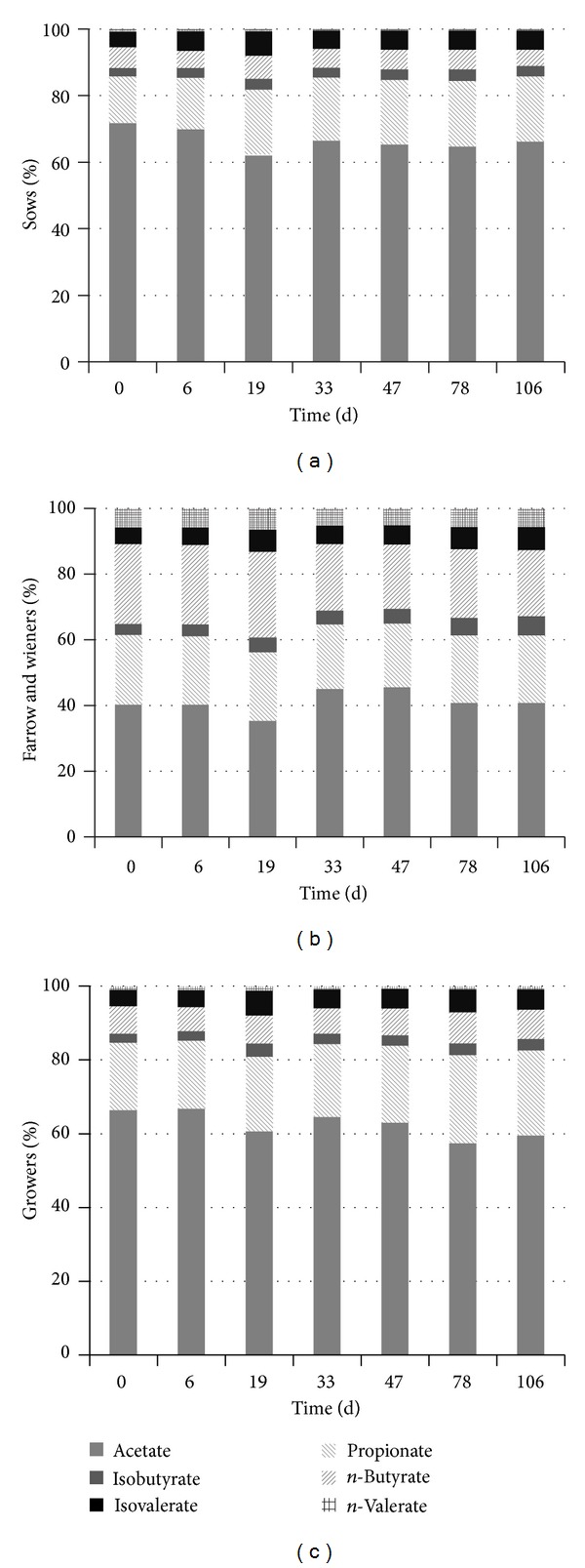
VFA composition during storage at 15°C in manure from Sows, Growers, and Farrow and Wieners.

The COD_VFA_ had start values of 3.2, 7.3, and 12.7 g L^−1^ and maximum values of 12.1, 20.2, and 21.5 g L^−1^, at days 106, 106, and 47 for Sows, Growers, and Farrow and Wieners, respectively. Similar increase in VFA has been observed by others [13]. Acetate, propionate, and butyrate constituted most of the COD_VFA  _ content (Figure 4).

**Figure 5:**
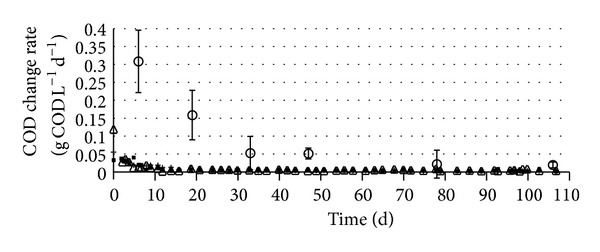
Methane production during storage at 15°C for Sows (■), Growers (∆), and Farrow and Wieners (+), and average change in CODs in the 3 manures (○) during storage at 15°C.

The pH started at 8.5, 7.8, and 6.5 for Sows, Growers, and Farrow and Wieners, respectively, dropping quickly to 7.1, 7.1, and 6.4 and staying rather constant for the whole test of 106 days (Figure 3). The soluble organics content (COD_S_) increased mainly during the first month (Figures 3 and 5), from 8.6, 15.3, and 18.7 g L^−1^ for Sows, Growers, and Farrow and Wieners, respectively, to 13.9 (61% increase), 21.6 (41% increase), and 21.0 (12% increase) after 33 days, with a maximum of 116, 25, and 23 g L^−1^ at 78, 78, and 47 days, respectively. This implies that one month pig manure storage prior to AD is favorable, assuming that COD_S_ roughly equals the AD methane production potential. Changes in TS, VS, NH_4_-N, and alkalinity concentrations during storage are small and within the range of standard deviations for these parameters (Table 1).

#### 3.2.2. Methane Loss

A disadvantage of long term manure storage is the potential for methane loss. Methanogenesis is however a slow process which can be inhibited by pH below 6.5 [14] and high free ammonia concentrations [15] and slowed down by reduced temperatures [8]. Methane production was detected throughout the 15°C laboratory storage test but the rate was close to zero (<0.01 g COD L^−1^d^−1^) except during the first week of storage (Figure 5). The methane loss is compared to the COD_S_ production rate in Figure 5. The COD_S_ production was higher than but dropping towards the methane loss. Real long storage time should be avoided to limit GHGE. The methane loss compared to the methane potential after 33 days is 1.7, 1.0, and 0.7% for Sows, Growers, and Farrow and Wieners, increasing to 2.4, 1.5, and 1.0%, respectively, after 78 days.

### 3.3. Full Scale Storage

**Figure 6:**
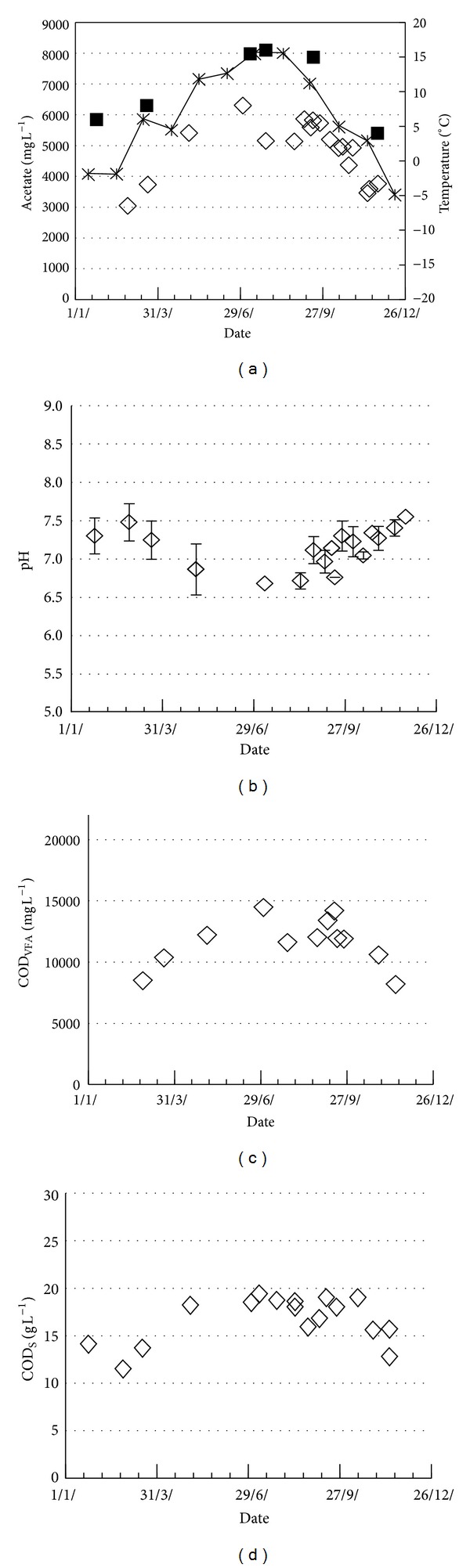
Acetate, COD_VFA_, pH, and COD_S_ in pig manure during basin storage at the farm. Average monthly air temperature (∗) and temperature in the basin (■).

The average monthly air temperatures varied between −5°C and +16°C and in the full scale manure storage basin between +4°C in December and +16°C in July (Figure 6) which is similar to outdoor storage temperatures reported from Sweden, Denmark, and Canada [16–18]. Concentrations of dissolved organics varied throughout the year in phase with temperature changes (Figure 6). Both total VFA and the acetic acid levels were nearly two times higher in the summer compared to the winter. This caused seasonal pH changes from 6.7 in summer to 7.4 in winter. The total content of soluble organics, CODs, did not change as much as the VFA, with values of 11–16 g L^−1^ during the coldest period and 14–19 g L^−1^ during spring, summer, and autumn (Figure 6). This suggests that disintegration and hydrolysis are less temperature dependent than acidogenesis during manure storage.

### 3.4. Implications

The study was mainly motivated by the idea that high rate AD reactors may give efficient manure treatment if it is well integrated with existing farm infrastructure for slurry based manure handling systems. The results confirm that such solutions are feasible: pig manure separates by gravity into layers where the main, middle layer is a substrate suitable for high rate AD. This substrate with 50 days average HRT in the full scale case investigated contains easily degradable organics, mainly VFA, at concentrations suitable for high rate AD.

The dissolved organics content in the full scale case (Figure 6) is approximately the same as the average concentrations in the laboratory experiments (Figure 3), implying that these small scale tests yield values realistic for full scale applications and more insight than obtainable in field studies. The laboratory tests suggest that the highest CODs concentration is obtained after 3 months storage with some variations among the different production stages, but where most of the CODs generation is achieved after 1 month. After 3 month storage the CODs generation by hydrolysis has decreased almost to the level of CODs loss by methanogenesis (Figure 5). This implies that the basement storage investigated has a manure retention time (50 d average) ideal for further AD to maximize methane production. During winter, however, little hydrolysis occurs at 4–6°C so that AD feed from this basin will have similar soluble organics concentrations as that of fresh manure towards the end of the winter. The methane production potential of the stored manure is thereby lowest when the farm heat demand is the highest. This is a disadvantage if the generated methane is used for heating purposes, but it may be compensated for by increasing the hydraulic loading rate of the AD.

This investigation is based on the observation that pig manure naturally separates into three layers with a middle layer with much less suspended solids than the floating and bottom layers (Figure 1) and the assumption that this middle layer is suitable as high rate AD feed. An extensive study to evaluate how suitable this manure middle layer is as feed for sludge bed AD is in progress. The results are not yet published but it is observed that biogas yield is closely related to both the CODs and VFA contents of the feed, implying that these measurements can be used to evaluate the methane potential of such manures. It is also observed that stable methane production (~5 L CH_4_ L^−1^ d^−1^) is obtained at 1 d HRT in lab scale UASB reactors with such manure as the only feed. The proposed concept of combining existing manure storage facilities with high rate AD has, therefore, potential to become a cost-effective individual farm solution for biogas generation.

Only the middle manure layer is used as AD feed in the concept evaluated here, implying that some of the total biogas potential is not directly utilized. Further investigations will be carried out to quantify and limit this loss. The following observations can be relevant for how much methane potential is not recovered by the HRAD approach evaluated here. (1) The top and bottom layers constitute less than 30% of total manure volume. (2) These two layers are kept in the storage for a long time (up to one year) during which disintegration and hydrolysis can transform particles in these layers to CODs that will diffuse into the middle layer and can thereby become part of the utilized AD substrate.

The strongest methane emission from manure occurs during the first hours after it is released from the animals, an effect that can probably not be prevented since it occurs in the barn. Some but very limited and quite constant methane release is observed after the first days of storage (Figure 5), implying that long term storage without or prior to AD for methane recovery will cause some GHGE. This emission must be included when determining optimal storage time prior to AD with respect to “carbon footprint” of such solutions. Optimal storage will, therefore, be less than 3 months during the warmer seasons. This can be achieved in the full scale case investigated by lowering manure HRT simply by changed manure handling routines, maintaining lower liquid level in the storage basin.

## 4. Conclusion

The amounts of easily degradable organics in pig manure depend on the source of the manure (production stage) and the storage time and temperature. Lab scale results correspond well with measurements from full scale storage of manure from the same barn. Temperature effects on generation of dissolved organics and methane during long term storage from lab tests are therefore useful to understand the processes occurring in farm storage basins.

Slight and quite constant methane emissions were detected through months of storage. The strongest methane emission occurred the first days and is therefore hard to avoid since that is when the manure is in transit from the animals to the storage.

Temperature significantly influenced manure quality during storage. Dissolved organics are generated by disintegration and hydrolysis of particles during storage in the warmest season (manure temperatures 10–15°C) but not at winter temperatures (4–6°C). The manure from the Farrow and Wieners stage, studied in more detail for temperature effects, gained no significant CODs increases.

Most of the dissolved organics are VFA, mainly acetate, and the ratios between the various VFAs remained quite constant for all conditions tested.

The production of dissolved organics showed some variations among manure from the different production stages. The increase of CODs at 15°C were 61%, 41%, and 12% for the three production stages Sows, Growers, and Farrow and Wieners, respectively, after one month.

Dissolved organics generation is highest initially and gradually drops to a low level during the first month at 15°C. The dissolved organics leveled off after three months storage (at 15°C), when the CODs production had dropped almost to the level of the methane production. The full scale basement storage has an average HRT of 50 d so most of the CODs generation potential can therefore be obtained using this storage as pretreatment for AD.

Pig manure separates by gravity into layers where the main, middle layer is a substrate suitable for high rate AD. It is therefore concluded that ordinary basement manure storage basins can be used to make feed with easily degradable organics, mainly VFA, at concentrations suitable for high rate AD. Efficient manure treatment for methane generation by high rate AD integrated with existing farm infrastructure for slurry based manure handling appears to be a promising option that warrants further investigation.

## Figures and Tables

**Table 1:** Average concentrations and standard deviations during storage at 15°C.

	Sows	Growers	Farrow and Wieners
TS (g L^−1^)	51.9 ± 2.5	78.7 ± 4.1	70.0 ± 3.2
VS (g L^−1^)	34.7 ± 2.1	54.7 ± 3.4	55.5 ± 2.7
NH_4_-N (g L^−1^)	2.6 ± 0.2	3.1 ± 0.3	1.9 ± 0.5
Alkalinity (g L^−1^)	15.9 ± 0.6	20.3 ± 1.4	9.8 ± 0.6
